# Casualties of peace: an analysis of casualties admitted to the intensive care unit during the negotiation of the comprehensive Colombian process of peace

**DOI:** 10.1186/s13017-017-0161-2

**Published:** 2018-01-16

**Authors:** Carlos A. Ordoñez, Ramiro Manzano-Nunez, Maria Paula Naranjo, Esteban Foianini, Cecibel Cevallos, Maria Alejandra Londoño, Alvaro I. Sanchez Ortiz, Alberto F. García, Ernest E. Moore

**Affiliations:** 1 Division of Trauma and Acute Care Surgery, Fundación Valle del Lili, Cali, Colombia; 2 Department of Surgery, Universidad del Valle, Cali, Colombia; 3 Clinical Research Center, Fundación Valle del Lili, Cali, Colombia; 4 School of Medicine, Universidad ICESI, Cali, Colombia; 5 Department of Surgery, Trauma Research Center, University of Colorado, Denver, CO USA; 6 Department of Surgery, Clinica Foianini, Santacruz de la Sierra, Bolivia

**Keywords:** Wounds and injuries, Military personnel, Peace, Casualties, Trauma, Critical care, Critical care outcomes

## Abstract

**Background:**

After 52 years of war in 2012, the Colombian government began the negotiation of a process of peace, and by November 2012, a truce was agreed. We sought to analyze casualties who were admitted to the intensive care unit (ICU) before and during the period of the negotiation of the comprehensive Colombian process of peace.

**Methods:**

Retrospective study of hostile casualties admitted to the ICU at a Level I trauma center from January 2011 to December 2016. Patients were subsequently divided into two groups: those seen before the declaration of the process of peace truce (November 2012) and those after (November 2012–December 2016). Patients were compared with respect to time periods.

**Results:**

Four hundred forty-eight male patients were admitted to the emergency room. Of these, 94 required ICU care. Sixty-five casualties presented before the truce and 29 during the negotiation period. Median injury severity score was significantly higher before the truce. Furthermore, the odds of presenting with severe trauma (ISS > 15) were significantly higher before the truce (OR, 5.4; (95% CI, 2.0–14.2); *p* < 0.01). There was a gradual decrease in the admissions to the ICU, and the performance of medical and operative procedures during the period observed.

**Conclusion:**

We describe a series of war casualties that required ICU care in a period of peace negotiation. Despite our limitations, our study presents a decline in the occurrence, severity, and consequences of war injuries probably as a result in part of the negotiation of the process of peace. The hysteresis of these results should only be interpreted for their implications in the understanding of the peace-health relationship and must not be overinterpreted and used for any political end.

## Background

After decades of a civil war that caused thousands of military and civilian victims [1, 2] in 2012, the Colombian Government started a process of peace with the left-wing guerrillas “Fuerzas Armadas Revolucionarias de Colombia” (FARC). The process of peace aimed to put a definitive end to the rural violence that plagued the country for more than a half century. To this end, both parties (government and guerillas) agreed to a truce that started in November 2012 [3].

Collective violence in the form of war is responsible for illness and death [4, 5]. It has been estimated that 191 million people died as a result of armed conflict during the twentieth century [4]. Among the many adverse effects of armed conflict, war-fighters carry a higher risk of suffering the immediate and deadly impact of war and military operations as they can be killed or injured on the battlefield [6]. Despite the catastrophic consequences of war on the health of populations, the majority of healthcare research in this field has been focused on analyzing the epidemiology of combat injuries on the battlefield during ongoing war periods [7–10]. However, there are no contemporary descriptions of hostile casualties during times of peace negotiation and implementation.

We hypothesized that the negotiation of the Colombian process of peace reduced the number of severe injuries and their consequences. As intensive care unit (ICU) admissions can provide an indirect measure of severe injuries, we sought to analyze casualties who were admitted to the ICU before and during the period of the negotiation of the comprehensive Colombian process of peace.

## Methods

### Study design

Following our hypothesis, a retrospective study was carried out to analyze casualties who were admitted to the ICU before and during the period of the negotiation of the comprehensive Colombian process of peace. According to the process of peace timeline, we set up two study periods: the first period was the one before the declaration of the truce [3] (January 2011–November 2012) and the second was the one after the truce (November 2012–December 2016: the negotiation period).

### Data source

For this retrospective observational study, we used data from the prospective Trauma Registry of the Panamerican Trauma Society. A description of this registry is found elsewhere [11, 12]. Data was complemented with information from medical charts**.** We reviewed data of hostile casualties admitted to the intensive care unit from 2011 to 2016 at La Fundación Valle del Lili (FVL) University Hospital, in Cali, Colombia. The FVL Institutional Review Board approved the study protocol.

FVL is a level I trauma center with 510 beds, 80 in adult intensive care units (ICUs), of which ten are reserved for trauma patients. It serves as a referral facility for both civilian and military trauma from the southwest region of the country. The FVL referral area for military trauma covers the southwest region of Colombia (131,301 km^2^), which corresponds to the departments of Nariño, Cauca, Valle del Cauca, and the southern part of Chocó. FVL is the only level I trauma center of the southwest region of the country with a partnership agreement with the Colombian Army for the care of the soldiers wounded in combat.

Historically, the FVL referral area (Southwest Region) has been the most affected by violence between guerrilla groups and military forces [13, 14].

### Patients

An initial screening of the Trauma Registry and FVL medical charts identified all soldiers admitted to the emergency room from January 2011 to December 2016. We were able to identify all soldiers because FVL electronic medical records contain data on the insurance company of each patient, and soldiers are the only ones that are covered by the “Dirección de Sanidad Militar” Colombian Army insurance. Furthermore, all hostile casualties are classified as “soldier wounded in combat” at the moment of arrival to the emergency room.

We included hostile casualties. Soldiers who were injured severely enough to be admitted to the intensive care unit were included. Soldiers who arrived at the ER but were not injured in action were excluded. A warfighter was classified as a hostile casualty if he was a victim of terrorist activity or became a casualty in action [15]. Soldiers wounded in combat (hostile casualties) were managed following institutional protocols by the same group of surgeons during the period observed.

We recorded data on demographics, trauma, and clinical characteristics, the severity of the injury, resuscitation and operative strategies, organ dysfunction, activation of the trauma transfusion protocol, and mortality.

We calculated organ dysfunction as defined by Vincent et al. [16] (SOFA Score). Patients who needed the activation of the trauma transfusion protocol were those who (1) died early from hemorrhage, (2) patients who, within 24 h of admission received ≥ 3 U PRBCs within 1 h, (3) patients who received massive transfusion (MT) (MT > 10 U in 24 h), and (3) patients with a hemorrhage that required plasma and platelet transfusion. Patients suffering severe trauma were those who had an Injury Severity Score (ISS) greater than 15.

### Statistical analysis

Descriptions of all patients were performed using relative and absolute frequencies for qualitative variables. Continuous variables were summarized by reporting medians and inter-quartile ranges. Variables of interest were compared between groups (group before the truce vs. group after the truce) using the Wilcoxon-Mann-Whitney *U* Test for continuous variables and the Fisher exact test for categorical variables. We considered multivariate regression analysis to asses for differences in severe trauma occurrence (ISS > 15) between periods. All analyses were conducted using Stata statistical software. A *p* < 0.05 was considered significant.

## Results

**Fig. 1:**
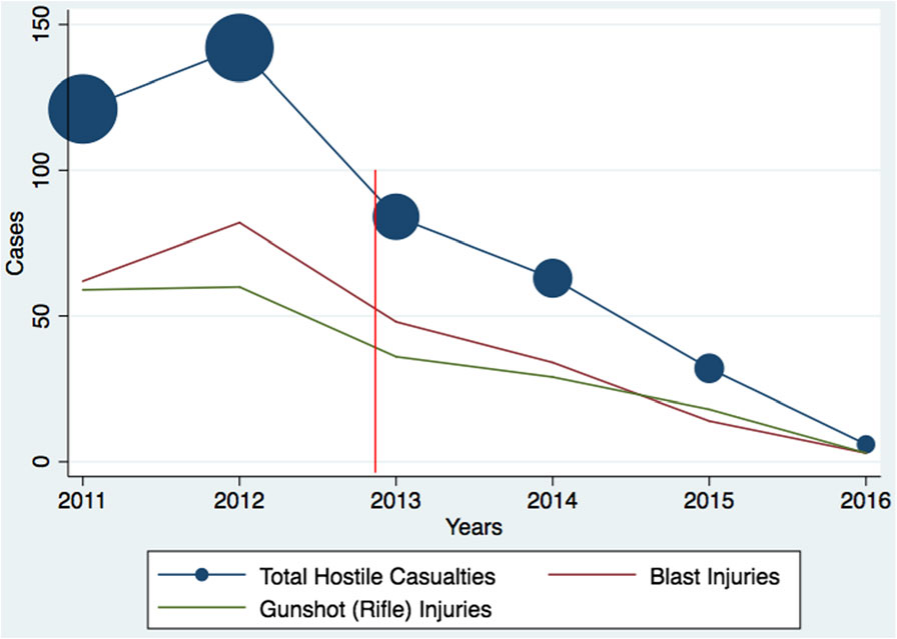
Variations in hostile casualties’ admissions and trauma mechanisms 2011–2016. (Description: shows the variations in hostile casualties admissions to the ER and trauma mechanisms during the period observed. Blue circles: graphic representation of the proportion of ICU admissions each year)

During the 6 years observed, 448 soldiers wounded in action were admitted to the emergency room. Figure 1 provides an overview of the variations in the number of hostile casualties admitted to the ER for the years 2011 to 2016. There was a gradual decrease in the number of warfighters admitted to the ER since the beginning of the truce. Moreover, the numbers of blast and gunshot rifle injuries also declined over this period. In 2012, 142 soldiers wounded in combat were admitted to the ER. This number decreased to 84, 63, 32, and 6 ER admissions in 2013, 2014, 2015, and 2016, respectively.

Of all casualties admitted to the ER during the period observed, 94 required ICU care. Sixty-five presented before the negotiation of the process of peace period and 29 during the negotiation period.

All patients were male, and half were less than 25 years old. Soldiers were victims of blast (52%) and gunshot rifle injuries (48%) in almost equal proportions. Twenty-five of those with blast injuries suffered land mine injuries. On admission, half of the patients had a shock index greater than 0.9 and one-third (31%) presented with hypotension (SBP < 90 mmHg). Soldiers suffered predominantly severe traumatic injuries. The median (IQR) Injury Severity Score was 20 (14–29), with 70% of patients scoring more than 15.

Differences in demographics, initial clinical variables, and injury severity by periods are presented in Table 1. There were no statistically significant differences between groups with respect to age, vital signs, mode of transportation to the ER, and mechanism of trauma. Injury Severity Scores were significantly higher in patients who presented before the truce than in patients who presented after [ISS, median (IQR): 25 (16-30) vs 14 (10-22); *p* < 0.01]. The proportion of patients presenting with an ISS greater than 15 was significantly higher before the truce (*p* < 0.01).

**Table 1:** Demographics, initial clinical variables, and injury severity with respect to periods

	Before the negotiation period (*n* = 65)	During the negotiation period (*n* = 29)	*p*
Age	25 (21–30)	26 (24–29)	0.41
SBP	110 (82–134)	116 (84–131)	0.98
Shock Index	0.90(0.67–1.3)	0.92(0.67–1.2)	0.99
HR	102 (84–120)	110 (88–120)	0.58
Tachycardia*, *n*	38	21	0.25
Hypotension**, *n*	22	8	0.63
Mode of transport			
Helicopter, *n*	53	25	0.76
Ambulance, *n*	12	4	0.76
Trauma mechanism			
Gunshot rifle, *n*	30	15	0.66
Blast injuries, *n*	35	14	0.66
Landmine injuries, *n*	16	9	0.61
Trauma severity			
ISS, median (IQR)	25 (16–30)	14 (10–22)	< 0.01
ISS > 15, *n* (%)	53 (81.5)	13 (44.8)	< 0.01

*SBP* systolic blood pressure, *HR* heart rate, *ISS* Injury Severity Score

*Heart rate > 90 bpm; **systolic blood pressure < 90 mmHg

As shown in Table 2, no significant differences were found between periods with respect to resuscitation strategies, operative interventions, multi-organ failure, and mortality. However, fewer patients required surgery, damage control laparotomy (DCL), and the activation of the trauma transfusion protocol in the negotiation period after the truce. Moreover, fewer amputations and ostomies (intestinal diversions) were performed during the period of peace negotiation. Furthermore, the occurrence of severe trauma and serious injuries gradually decreased in the period of peace negotiation (Table 3).

**Table 2:** Resuscitation and operative strategies, multi-organ failure, and mortality with respect to periods

	Before the negotiation period (*n* = 65)	During the negotiation period (*n* = 29)	*p*
PRBCs *	2 (0–4)	2 (0–3)	0.27
Crystalloids *	5050 (3350–7650)	4400 (3350–6400)	0.35
Required activation of the TTP	28	10	0.49
Required surgery, *n*	61	26	0.67
DCL, *n*	14	7	0.79
Ostomies, *n*	10	1	0.16
Amputations, *n*	13	5	1
SOFA score *	3 (1–7)	3 (1–7)	0.97
Organ dysfunction			
Renal dysfunction, n	9	9	0.08
Hemodynamic dysfunction, *n*	14	8	0.6
Coagulopathy, *n*	41	20	0.8
Respiratory dysfunction, *n*	48	17	0.13
Organ failure			
Cardiovascular failure, *n*	13	8	0.5
Coagulation failure, *n*	6	2	1
Respiratory failure, *n*	16	7	1
Mortality, *n*	1	0	0.9

*PRBCs* packed red blood cells, *TTP* Trauma Transfusion Protocol, *DCL* damage control laparotomy

*Median (IQR)

**Table 3:** Injuries, ostomies, and amputations per year

Year	2011 (*n* = 32)	2012 (*n* = 33)	2013 (*n* = 13)	2014 (*n* = 9)	2015 (*n* = 6)	2016 (*n* = 1)
Severe trauma (ISS > 15), *n*	28	25	9	0	3	1
Serious extremity Injuries*, *n*	19	11	7	1	1	0
Serious chest Injuries*, *n*	5	8	1	3	0	0
Serious abdominal Injuries*, *n*	7	17	2	2	1	0
Ostomies, *n*	3	7	1	0	0	0
Amputations, *n*	9	4	4	0	1	0

*ISS* Injury Severity Score

*Serious injuries: AIS ≥ 3

**Fig. 2:**
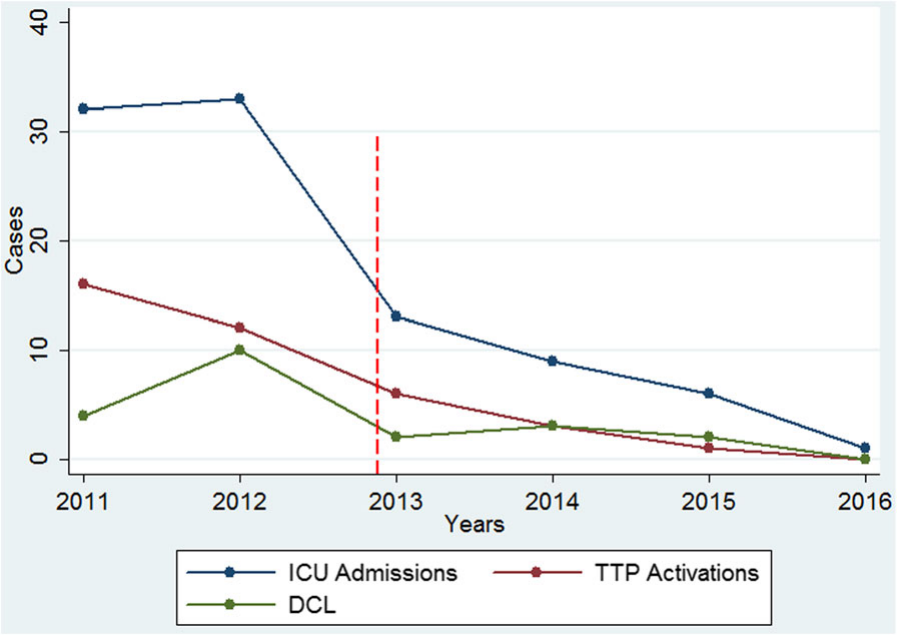
Variations in ICU admissions Trauma Transfusion Protocol Activations (TTP) and Damage Control Laparotomy. (Description: shows the variations in ICU admissions and the performance of damage control resuscitation procedures each year (TTP, Trauma Transfusion Protocol (red line); DCL, damage control laparotomy (green line); ICU; intensive care unit (blue line))

As shown in Fig. 2, there was a considerable reduction in the performance of procedures related to trauma care during the period observed. In 2012, 33 patients were admitted to ICU, ten required damage control laparotomy, and 12 underwent the application of the trauma transfusion protocol. These numbers decreased to three DCL, three Trauma Transfusion Protocol (TTP) activations, and nine ICU admissions in 2014, then dropped to zero DCL, zero TTP activations, and one ICU admission in 2016.

Finally, the odds of suffering severe trauma were higher in patients who presented before the truce than in patients who presented after the negotiation period (unadjusted OR = 5.4; (95% CI, 2.0–14.2); *p* < 0.01). Although we considered multivariate regression analysis to assess for differences in severe trauma occurrence between periods, the only significant difference that we found in univariate analysis was for injury severity, and thus, we decided to present the unadjusted odds ratio for severe trauma occurrence.

## Discussion

To our knowledge, this is the first known analysis of warfighters requiring ICU care during a period of peace negotiation. We found that casualties presenting before the negotiation period were more likely to suffer severe trauma. Moreover, we describe a gradual decrease in the admission of hostile casualties to the ICU, the performance of damage control resuscitation and surgical procedures, and the consequences related to military trauma during the period observed. This number started to decline from 2013 onwards.

A total of 448 hostile casualties were registered of which 20% were injured severely enough to require ICU admission. Previous descriptions of critical care provision in austere war environments reported higher volumes of patients admitted to the ICU in shorter time periods [17, 18]. The first description of contemporary ICU data from the Operation Iraqi Freedom [18] reported the 1-month experience in the care of 47 critically ill patients. Our series report a 6-year experience in the care of 94 war fighters injured in combat. Although the volume of patients admitted to the ICU in our series seems to be smaller, this is likely to be a consequence of the characteristics of the armed conflict. Colombian conflict was below conventional war and above the routine peaceful competition between the state and independent groups. In this conflict, military operations were deployed to confront subversion (FARC) and high-velocity weaponry or high-order explosives were regularly used.

War has a deadly impact on human health [4]. Many people, but especially soldiers can suffer the direct consequences of war. The existing literature on war injuries is extensive and consistently describes increasing numbers of warfighters suffering war injuries [6–10]. For example, the Colombian armed forces reported that for the years 2005–2010 the number of soldiers wounded in combat increased and this was accompanied by an increase in the number of service members suffering severe trauma [19, 20]. In contrast, this study shows a gradual decrease in the admission warfighters to the ICU, the procedures related to trauma care, and the surgical interventions performed (damage control surgery, ostomies, and amputations). Furthermore, the number of patients suffering severe trauma (ISS > 15) and/or serious injuries (AIS ≥ 3) gradually decreased during the period observed, and thus, we present a pattern that has not previously been described.

In the context of collective violence in the form of war, the severity of injuries suffered by warfighters could be seen as a measure of the intensity of the war. Therefore, the reduction in the proportion of warfighters suffering severe injuries could be a proxy indicator for the positive impact of peace negotiation on war casualties. Regarding the latter, the current study found that there was a significant difference in average Injury Severity Scores between periods. Moreover, the odds of presenting with severe trauma were higher before the negotiation period. The reason why the proportion of warfighters suffering severe trauma was significantly lower during the negotiation period seems to be clear. The truce established in November 2012 was an agreement between the Colombian government and the FARC to stop hostile actions. During this truce, the efforts made by the leaders of both parties and their commitment to preventing the conflict from resuming, although not directly, inferred in reducing the number of injuries and associated consequences that we saw. On the other hand, the reason why average injury severity scores differed between periods is unclear. It could be that during the negotiation period, hostile actions were performed with less lethal weapons not involving classical high-velocity weaponry or high-order explosives, and thus, the nature of anatomical damage in soldiers’ victims of hostile acts in the late period could differ from that of warfighters in the time before the truce.

The number of casualties admitted to the ICU decreased gradually during the period observed, and this was accompanied by a reduction in the activations of the trauma transfusion protocol and the number of damage control surgeries performed (Fig. 2). Damage control resuscitation (DCR) is a structured intervention that includes early blood product transfusion, early hemorrhage control by damage control surgery, and restoration of physiologic stability [21, 22]. These interventions could have a great impact on resource utilization as patients managed following principles of DCR may require a longer intensive care unit and hospital stay and, thus, higher medical resources and costs. Traditionally, combat troops have required specialized medical resources for the resuscitation and the management of combat related injuries. As war increases its intensity, there is a proportional increase in the need for the deployment of more advanced medical capabilities to increases the odds of survival of the wounded in combat [23]. Therefore, war is not only a social catastrophe but also an economic one, and thus, our findings could go beyond the immediate reduction in the number of soldiers suffering trauma and requiring advanced trauma care and could have significant policy implications. It could be that in the future period of peace, advanced medical capabilities for the care of the wounded in combat will no longer be required and these resources could be used in civilian populations.

In the study that we present, the situation after (negotiation period) may differ in respects other than the exposure to peacebuilding if compared to the situation before (war period). For example, indications for laparotomy or resuscitation strategies such as transfusion protocols may vary over time [24, 25], and these variations could affect procedures rates and patient outcomes. Therefore, one can argue that the evolution and improvements in trauma care may be responsible in part for the differences observed. However, all included patients were treated by the same group of well-trained trauma surgeons following institutional protocols. Furthermore, during the study period, no significant improvements in the principles of trauma surgery and trauma care were developed, and thus, we believe that no other factors different from the process of peace could plausibly have caused any observed change in outcomes. Therefore, it is very probable that the period of peace negotiation was responsible in part for the pattern observed, and thus, our description, although limited, is an advance in the understanding of the beneficial effects of peace in the health of populations [26, 27].

Finally, although the Colombian armed conflict only delivered suffering and destruction, we must acknowledge how civilian trauma was positively affected by the experience of the war. Our hospital is located in the largest city of southwest Colombia and was initially conceived as a large non-profit, high-complexity center dedicated to the people of the southwest region of Colombia. However, as war increased its intensity, our center became a mixed military-civilian center, and we had to provide surgical critical care to the wounded in combat. We learned how to perform simple surgical maneuvers to optimize times during damage control surgery [28–30] and thus be able to control contamination and stop the bleeding on time. Therefore, it is likely possible that competencies acquired from warfighters were translated to daily practice and played a key role in improving outcomes of injured patients in the civilian sector.

### Limitations

Although our hospital is the only referral level I trauma center for military casualties in the southwest region of Colombia, we can only have access to casualties admitted to the emergency department. Moreover, we had no data of casualties from other areas of the country and those who died before reaching a medical facility; all of which may have introduced significant selection and information bias. However, other reports also have shown a reduction in the number emergency department admissions of soldiers wounded in combat in different regions of the country probably as a result of the period of peace negotiation [31, 32]. For example, commenting on the effects of the process of peace on military casualties, the director of the Central Military Hospital argues [33]: *“I see very positive changes. Saving one life is already a very big win, but we went from having about 400-500 amputees [a year] to, this year, having much fewer cases. Over the course of the year (2017), we have had about 19 injured”.*

Further selection bias could be introduced as we only included soldiers admitted to the ICU. However, we wanted to have a description and analysis of a homogeneous population suffering severe trauma. Therefore, we decided to include soldiers wounded enough to require ICU care as this could be an indirect measure of severe trauma. Finally, the methodology used does not provide substantial evidence about the positive effect of peace negotiation on war casualties. However, our results are of importance for advancing the knowledge of peace-health relationship [26, 27] and for achieving the universal desire for peace.

Despite these results, questions remain, and future studies should investigate the long-term outcomes of severely wounded warfighters in the post-conflict period. Also, additional explorations of the effect of peace on civilian trauma should be undertaken. Finally, further multicentric studies with bigger sample sizes and which uses other designs such as time series analysis will need to be conducted to support our initial observations.

## Conclusion

We describe a series of war casualties who required ICU care in a period of peace negotiation. Despite our limitations, our study presents a decline in the occurrence, severity, and consequences of war injuries probably as a result in part of the negotiation of the process of peace. The hysteresis of these results should only be interpreted for their implications in the understanding of the peace-health relationship and must not be overinterpreted and used for any political end.

## References

[1] RNI. Registro Único de Víctimas (Colombian Victims Registry) [Internet]. Available from: http://rni.unidadvictimas.gov.co/RUV Accessed 11 Jan 2017.

[2] Rubiano AM, Sánchez ÁI, Guyette F, Puyana JC (2010). Trauma care training for National Police Nurses in Colombia. Prehosp Emerg Care.

[3] BBC. Colombia: FARC declares unilateral truce at landmark talks [Internet]. BBC news. 2012 [cited 2017 Jan 11]. Available from: http://www.bbc.com/news/world-latin-america-20399152. Accessed 11 Jan 2017.

[4] Sidel VW, Levy BS (2008). The health impact of war. Int J Inj Control Saf Promot.

[5] Haagsma JA, Graetz N, Bolliger I, Naghavi M, Higashi H, Mullany EC, et al. The global burden of injury: incidence, mortality, disability-adjusted life years and time trends from the Global Burden of Disease study 2013. Inj Prev. 2015;22:3–18. Available from: http://injuryprevention.bmj.com/content/early/2015/10/20/injuryprev-2015-041616 10.1136/injuryprev-2015-041616 PMC4752630 26635210

[6] Gawande A (2004). Casualties of War—military care for the wounded from Iraq and Afghanistan. N Engl J Med.

[7] Eastridge BJ, Mabry RL, Seguin P, Cantrell J, Tops T, Uribe P, et al. Death on the battlefield (2001–2011): Implications for the future of combat casualty care. J Trauma Acute Care Surg. 2012;73. Available from: http://journals.lww.com/jtrauma/Fulltext/2012/12005/Death_on_the_battlefield__2001_2011___.10.aspx 10.1097/TA.0b013e3182755dcc 23192066

[8] Belmont PJJ, Goodman GP, Zacchilli M, Posner M, Evans C, Owens BD. Incidence and Epidemiology of Combat Injuries Sustained During “The Surge” Portion of Operation Iraqi Freedom by a U.S. Army Brigade Combat Team. J Trauma Acute Care Surg. 2010;68. Available from: http://journals.lww.com/jtrauma/Fulltext/2010/01000/Incidence_and_Epidemiology_of_Combat_Injuries.35.aspx 10.1097/TA.0b013e3181bdcf95 20065776

[9] Kelly JF, Ritenour AE, McLaughlin DF, Bagg KA, Apodaca AN, Mallak CT, et al. Injury Severity and Causes of Death From Operation Iraqi Freedom and Operation Enduring Freedom: 2003–2004 Versus 2006. J Trauma Acute Care Surg. 2008;64. Available from: http://journals.lww.com/jtrauma/Fulltext/2008/02001/Injury_Severity_and_Causes_of_Death_From_Operation.6.aspx 10.1097/TA.0b013e318160b9fb 18376168

[10] Antebi B, Benov A, Mann-Salinas EA, Le TD, Cancio LC, Wenke JC (2016). Analysis of injury patterns and roles of care in US and Israel militaries during recent conflicts: two are better than one. J Trauma Acute Care Surg.

[11] Ordoñez CA, Morales M, Rojas-Mirquez JC, Bonilla-Escobar FJ, Badiel M, Miñán Arana F, et al. Trauma Registry of the Pan American Society of Trauma: one year of experience in two referral centers in the Colombian southwestern. Colomb Médica. 2016;47(3) PMC5091273 27821894

[12] Ramachandran A, Ranjit A, Zogg CK, Herrera-Escobar JP, Appelson JR, Pino LF, et al. Comparison of epidemiology of the injuries and outcomes in two first-level trauma centers in Colombia using the Pan-American Trauma Registry System. World J Surg. 2017:1–7. 10.1007/s00268-017-4013-8 28417184

[13] Bergquist CW, Peñaranda R, Sánchez G. G. Violence in Colombia, 1990-2000 : waging war and negotiating peace. Lanham, MD: Rowman & Littlefield; 2001.

[14] Glade J. Drug, guerrilla violence is crippling Colombia’s southwestern Cauca department. Colomb Reports. 2011. Available from: https://colombiareports.com/drug-guerrilla-violence-is-crippling-cauca/. Accessed 11 Jan 2017.

[15] United States Department of Defense. Department of Defense Dictionary of Military and Associated Terms. US Dep Def Jt Publ. 2015. Available from: http://www.dtic.mil/doctrine/new_pubs/jp1_02.pdf. Accessed 11 Jan 2017.

[16] Vincent JL, Moreno R, Takala J, Willatts S, De Mendonça A, Bruining H, et al. The SOFA (Sepsis-related Organ Failure Assessment) score to describe organ dysfunction/failure. Intensive Care Med. 1996;22:707–10. 10.1007/BF01709751 8844239

[17] Lundy JB, Swift CB, McFarland CC, Mahoney LCP, Perkins RM, Holcomb JB. A descriptive analysis of patients admitted to the intensive care unit of the 10th combat support hospital deployed in Ibn Sina, Baghdad, Iraq, from October 19, 2005, to October 19, 2006. J Intensive Care Med. 2010;25:156–62. Available from: http://journals.sagepub.com/doi/abs/10.1177/0885066609359588?journalCode=jica 10.1177/0885066609359588 20097667

[18] Lockey DJ, Nordmann GR, Field JM, Clough D, Henning JDR (2017). The deployment of an intensive care facility with a military field hospital to the 2003 conflict in Iraq. Resuscitation.

[19] Camargo J, Pérez LE, Franco C, Rodríguez E, Sánchez W. “Plan pantera”, trauma militar en Colombia. Rev Colomb Cir. 2014;29:293-304. Available from: http://www.scielo.org.co/pdf/rcci/v29n4/v29n4a5.pdf

[20] Arias C, Villamil E, Gutierrez J, Morales H, Sanchez W (2012). Trauma vascular periférico de guerra en Colombia: análisis epidemiológico de ocho años. Rev Colomb Cirugía.

[21] Mizobata Y. Damage control resuscitation: a practical approach for severely hemorrhagic patients and its effects on trauma surgery. J Intensive Care. 2017;5:–4. Available from: 10.1186/s40560-016-0197-5 10.1186/s40560-016-0197-5 PMC8600903 34798697

[22] Holcomb JB. Damage Control Resuscitation. J Trauma Acute Care Surg. 2007;62(6 Suppl):S36-7. Available from: http://journals.lww.com/jtrauma/Fulltext/2007/06001/Damage_Control_Resuscitation.29.aspx 10.1097/TA.0b013e3180654134 17556961

[23] Patel TH, Wenner KA, Price SA, Weber MA, Leveridge A, McAtee SJ. A U.S. Army Forward Surgical Team’s Experience in Operation Iraqi Freedom. J Trauma Acute Care Surg. 2004;57. Available from: http://journals.lww.com/jtrauma/Fulltext/2004/08000/A_U_S__Army_Forward_Surgical_Team_s_Experience_in.1.aspx 10.1097/01.ta.0000133638.30269.38 15345962

[24] ME K, LZ K, Narayan R, Al E (2013). A paradigm shift in trauma resuscitation: evaluation of evolving massive transfusion practices. JAMA Surg.

[25] Brinck T, Handolin L, Lefering R (2015). The effect of evolving fluid resuscitation on the outcome of severely injured patients: an 8-year experience at a tertiary trauma center. Scand J Surg.

[26] Joshi M (2015). Comprehensive peace agreement implementation and reduction in neonatal, infant and under-5 mortality rates in post-armed conflict states, 1989–2012. BMC Int Health Hum Rights.

[27] Gaffar AM, Mahfouz MS. Peace impact on health: population access to iodized salt in south Sudan in post-conflict period. Croat Med J. 2011;52:178–82. Available from: https://www.ncbi.nlm.nih.gov/pmc/articles/PMC3083250/10.3325/cmj.2011.52.178 PMC3083250 21495201

[28] Ordoñez CA, Pino LF, Badiel M, Sánchez AI, Loaiza J, Ballestas L (2011). Safety of performing a delayed anastomosis during damage control laparotomy in patients with destructive colon injuries. J Trauma.

[29] Garcia A, Martinez J, Rodriguez J, Millan M, Valderrama G, Ordoñez C (2015). Damage control techniques in the management of severe lung trauma. J Trauma Acute Care Surg.

[30] Ordoñez CA, Parra MW, Salamea JC, Puyana JC, Millán M, Badiel M (2013). A comprehensive five-step surgical management approach to penetrating liver injuries that require complex repair. J Trauma Acute Care Surg.

[31] CERAC. Assessing the merits of an imperfect peace: the FARC’s unilateral ceasefire in 2013-14. Blog CERAC. 2014. Available from: http://blog.cerac.org.co/assessing-the-merits-of-an-imperfect-peace-the-farcs-unilateral-ceasefire-in-2013-14. Accessed 11 Jan 2017.

[32] “Hace 51 años no se presentaba una reducción tan grande del conflicto armado”: Cerac. El Espectador [Internet]. 2016; Available from: https://www.elespectador.com/noticias/politica/hace-51-anos-no-se-presentaba-una-reduccion-tan-grande-articulo-611701. Accessed 11 Jan 2017.

[33] Daniels JP (2017). Frontline: caring for soldiers after the peace deal in Colombia. Lancet.

